# Collectively Facilitated Behavior of the Neonate Caterpillars of *Cactoblastis cactorum* (Lepidoptera: Pyralidae)

**DOI:** 10.3390/insects7040059

**Published:** 2016-10-31

**Authors:** Terrence D. Fitzgerald, Michael Wolfin, Ryan Young, Katelyn Meyer, Elizabeth Fabozzi

**Affiliations:** 1 Department of Biological Sciences, State University of New York, Cortland, NY 13045, USA; ryan.young02@cortland.edu (R.Y.); katelyn.meyer@cortland.edu (K.M.); ekfabozzi@gmail.com (E.F.); 2 Barton Lab, Department of Entomology, Cornell University, 630 West North Street, Geneva, NY 14456, USA; msw266@cornell.edu

**Keywords:** cactus moth, *Cactoblastis cactorum*, behavior, neonates, larvae, social caterpillar

## Abstract

The behavioral biology of the first instar larva of *Cactoblastis cactorum* was studied from the time of eclosion until the colony penetrated and initiated excavation of the host plant. Hatching from an egg stick was asynchronous, requiring 20 h for the entire cohort to eclose at 50%–70% RH and significantly longer at a lower range of RHs. On eclosion, neonates aggregated in an arena at the base of their egg stick and did not attempt to excavate the cladode until an average of 25 caterpillars had collected, approximately 15 h after the onset of egg hatch. Typically only a single entrance hole was formed, limiting the active process of excavating to one or a few individuals at-a-time until the host was fully penetrated and enlarged internally. Olfactometer tests showed that the neonates are strongly attracted to volatile chemicals released when caterpillars chewed into the cladode, accounting for the strong fidelity of the whole cohort to the initial site of penetration. In one instance, the caterpillars were observed to deal with an explosive release of mucilage by imbibing the liquid until the flooded zone was drained and the caterpillars could reenter the plant through the original entrance hole. Once inside the cladode, marked individuals adopted a regular cycle of defecating at the surface at a mean interval of approximately 10 min when followed for 35 successive cycles. Blanket spraying cladodes with a mandibular gland extract prior to hatching led to the independent dispersal of neonates and a failure to form an arena. When the cladode was impenetrable at the site of eclosion, the active cohort of unfed neonates set off together in search of a new site, marking and following a persistent trail that allowed late-to-eclose caterpillars to join their departed siblings. The adaptive significance of these observations is discussed in the context of the life history of the caterpillar.

## 1. Introduction

The caterpillar of *Cactobastis cactorum* is widely known for its ability to colonize cactuses and much of the technical literature on the insect has focused on either the economic impact of the insect on the host plant or aspects of the biology of the larva and adult relevant to its impact on the host. What we know of the basic behavioral biology of the caterpillar is built upon early field studies by Dodd [1] in Australia and Pettey [2] in South Africa. The caterpillar ecloses from an egg stick attached to the surface of a cactus cladode, which typically contains 60–100 eggs [3], though other investigators have reported averages of fewer eggs [4]. The neonates aggregate at the base of the egg stick under a loosely spun silken web then penetrate the cladode, creating an entrance hole. Typically, the whole colony enters the plant through this single entrance site, though several closely spaced entrance holes may be constructed [2]. Once inside, the neonates tunnel through the plant, returning to the surface to deposit waste in a debris field that encircles the entrance. More recent studies by [5,6,7,8,9] and others, as discussed later in the present paper, have provided additional details of the behavior of the neonates. Collectively, these studies show that the neonates are tightly bonded and suggest that they engage in suites of behaviors that are socially facilitated. As defined by Wilson [10], social facilitation occurs when a pattern of behavior is initiated or the pace or frequency of the pattern increases as a consequence of the presence or actions of another individual.

The study reported here was undertaken to fill in gaps in our knowledge of the behavioral biology of the neonates, from eclosion to the initiation of tunneling, and to investigate the importance of social interaction in facilitating their successful colonization of the host plant. To this end, we conducted investigations of: (1) the time course of the eclosion process; (2) the temporal relationship between the initiation of eclosion and the initiation of excavation; (3) the number of neonates required to successfully penetrate the cladode and the mean number of neonates assembled when excavation is initiated; (4) the proportional involvement of siblings in the task of establishing the entrance site; (5) the deposition of waste when tunneling; (6) the role of host volatiles in attracting the neonates to the excavation site; (7) the role of pheromones in containing the aggregate to the base of the egg stick and; (8) collective trail marking when the unfed cohort is forced to move to a new site. While some aspects of these studies have been undertaken by previous investigators, as will be discussed below, our methodology, and often our results, differed, lending additional insight into the behavioral ecology of the neonate caterpillars.

## 2. Experimental Section

### 2.1. Insects

Eggs of *C. cactoblastis* were obtained either from the U.S. Department of Agriculture Research Service, Crop Protection and Research Laboratory, Tifton, GA, USA or by breeding adults in the isolation facility at Cortland. Experiments involving host materials utilized spineless varieties of *Opuntia ficus-indica*.

### 2.2. Rate of Eclosion

A study was conducted to determine the extent to which the hatching of eggs within an egg stick is synchronized. An egg stick that was near to hatching was attached by its base to a paper card with hot glue in a natural, upright position. The egg stick was continually back-illuminated with fluorescent lighting to aid in the visualization of the eclosion process. Hatching was video recorded in time lapse at the rate of one frame per five second interval. Recording continued until no more hatching occurred. Date and time stamps were incorporated into the video recordings. The study was replicated with six different egg sticks having 51.2 ± 2.1 eggs. Temperature was maintained at 21 ± 3 °C and RH varied between approximately 50%–75% during the course of the study.

### 2.3. Eclosion During the Photophase and Scotophase of Light Cycle

To determine if there was a difference in the rate of egg hatch during the day and night, egg sticks were held at 21 ± 3 °C under a 14:10 L:D cycle for a minimum of seven days before they began to hatch and the rate of eclosion during the photophase and scotophase of the light cycle compared. The light regime was established with a fluorescent lamp for the light phase illumination and an IR LED lamp for the dark phase. Seven egg sticks with caterpillars near to hatching were each attached with hot glue in a natural upright position to separate cladodes and the hatching process video recorded in time lapse at the rate of one frame per five second interval. Recording continued until all of the eggs had hatched. The experiment was conducted in an open room where the temperature was maintained at 21 ± 3 °C but RH was not controlled and varied between 15% and 30% during the course of the study.

### 2.4. Effect of RH on Egg Hatch

A marked difference in the rate of egg hatching when egg sticks were held at the lower and higher RH’s in the above studies prompted an additional controlled study to assess the effect of RH on the rate and percentage of egg hatch. All egg sticks used in this study were held at 76% RH at 21 ± 3 °C up until the time of the study and at least 48 h elapsed between the time they were placed under the treatment RH and the eclosion of the first caterpillars. For the first experiment, egg sticks containing approximately 60 eggs were divided into three equal sections of 18–20 eggs and each section placed in a sealed petri plate at a randomly assigned RH for the duration of the hatch. RH’s were established with distilled water (100%) or with saturated salt solutions at 76% and 54% [11]. RH’s were monitored with an Amprobe THWD-5 meter (Amprobe, Melrose, MA, USA). The time-sequence of eclosion was video recorded at 1 frame/5 s with real time stamped into each video frame. The experiment was replicated with 8 different egg sticks. The time elapsed between the first and last egg to hatch was calculated for each treatment. A second experiment using the identical protocol but at a lower range of RH’s was conducted with sections of egg sticks held at 54%, 33% and 10% RH established with saturated salt formulations [11], and replicated five times at each RH with different egg sticks.

### 2.5. Aggregation and the Initiation of Excavation

**Figure 1:**
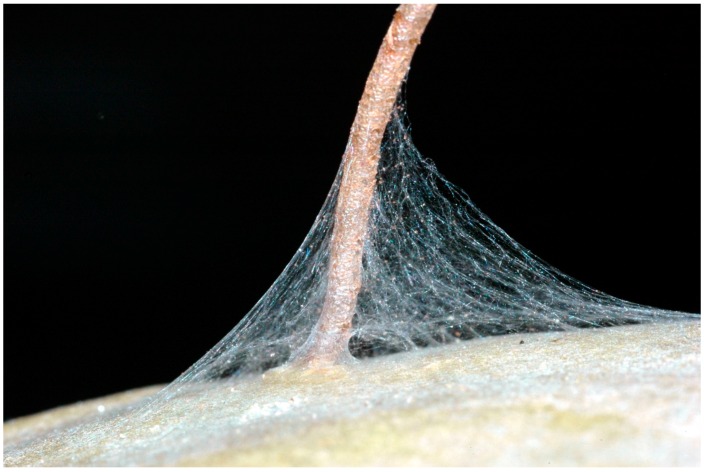
Silk covered arena formed by neonate *Cactoblastis cactorum* caterpillars at the base of the egg stick.

As previously described by other investigators, the neonates spin a web of silk at the base of the egg stick under which they aggregate before they begin to excavate the plant (Figure 1) [1,2]. Observations were made of the seven egg sticks used in the L:D experiment detailed above to determine: (1) the number of caterpillars aggregated at the base of the egg stick when excavation was initiated; (2) the time elapsed between the initiation of eclosion and the initiation of excavation; (3) the time required for the caterpillars to excavate to the depth of one full body length; and (4) the distance between the base of the egg stick and the entrance hole.

### 2.6. Ability of Individual and Small Groups of Caterpillars to Penetrate a Cladode

**Figure 2:**
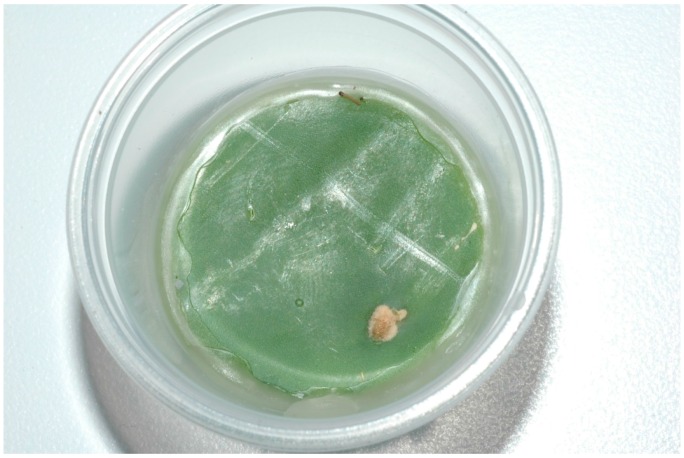
Container with section of cladode and single caterpillar. Top removed for clarity.

Circular disks, 32 mm diameter, were cut from cladodes and placed at the bottoms of 20 mL capacity transparent plastic portion-control containers (Figure 2). Each cactus disk was sealed to the sides of the container along its circumference with hot wax to prevent the disk from desiccating and the caterpillars from gaining easy access to the interior of the cactus from the cut edge. Care was taken to assure that the exposed surfaces of the disks were intact, lacking punctures that might allow the caterpillars to access the interior. A total of 61 of these containers were established and a single unfed and newly hatched *C. cactoblastis* caterpillar placed in each. Transparent tops were placed on the containers to seal them and the behavior of the caterpillars observed intermittently over a 48 h period to determine if they were successful in entering the plant. Using the same experimental setup, groups of five newly hatched and unfed caterpillars were placed in each of 13 additional containers and similarly observed. A small drop of water was placed near the caterpillars in both experimental setups at the initiation of the study. The caterpillars readily imbibed the water.

### 2.7. Orientation of Neonates to Host Volatiles

**Figure 3:**
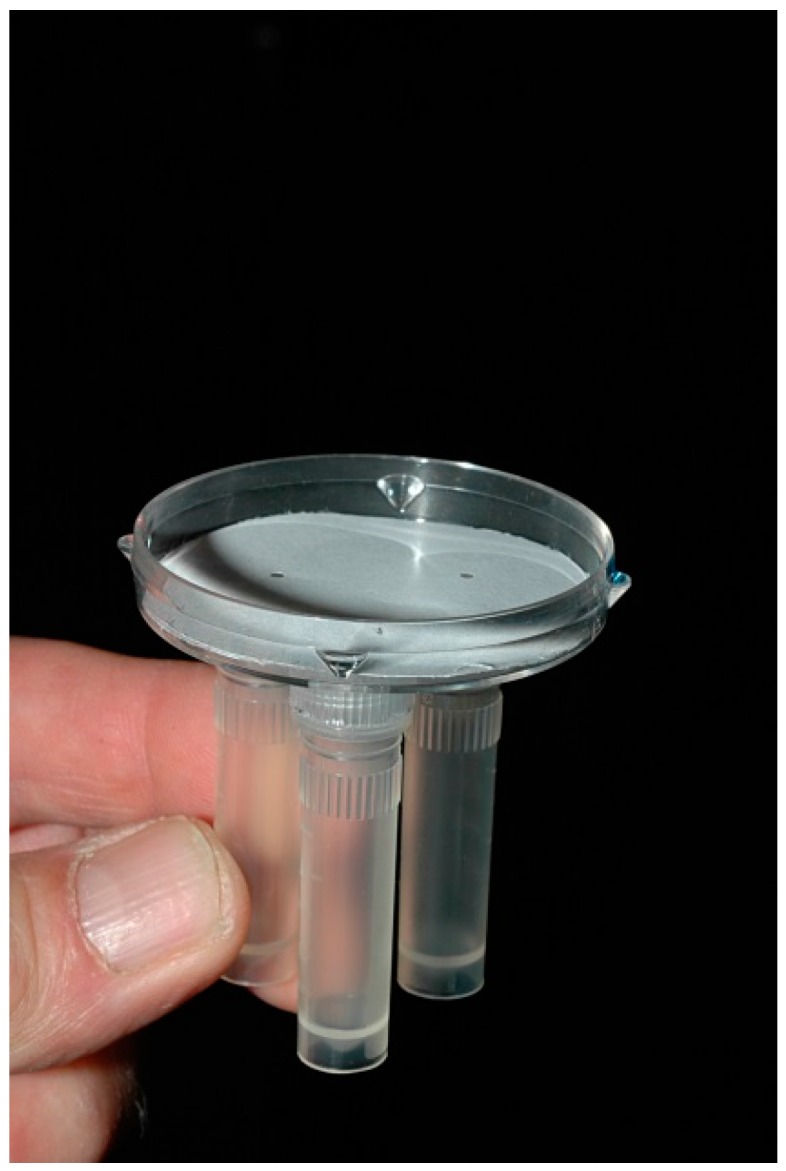
Olfactometer used to assess the response of neonates to cactus volatiles. See also Video S3.

Observations suggested that the newly eclosed, unfed caterpillars are strongly attracted to the entrance hole being excavated by the caterpillars. A study was conducted to determine if the caterpillars are attracted to the opening itself, water vapor emanating from the opening or to volatile chemicals released when the surface of the cladode is ruptured. An olfactometer was constructed as illustrated in Figure 3. Three holes arranged in a triangular pattern, each 6-mm in diameter, were drilled into the bottom of a 5-cm diameter petri plate. Holes of the same diameter were drilled though the centers of the screw tops of three 1-cm diameter by 4.5-cm long vials. The holes in the caps were aligned with the holes in the petri plate and the caps hot glued onto the plate. A small piece of cotton was placed near the top of each vial but below the surface opening to allow caterpillars that entered the hole easy access to the interior. In one vial the cotton was left dry, in another saturated with distilled water and in another placed loosely over slivers of cactus cut fresh from a cladode. The vials were each screwed into a randomly selected screw-top. To conduct a trial, a circular sheet of paper with three, 1.6-mm diameter openings aligned with those of the petri plate was placed in the bottom of the dish. A variable number of first or second instar caterpillars was then place in the dish and the plate closed. The response of caterpillars to the three openings was video recorded in real time. A positive response occurred when a caterpillar entered the opening and moved into the interior of a vial. The experiment was replicated eight times, each with different caterpillars and new preparations of the treatments. Between trials, the olfactometer was thoroughly washed with both ethanol and water rinses. New paper liners and new vials were used for each replicate of the test.

### 2.8. Group Excavation Behavior

The entire colony of caterpillars typically enters a cladode through a single hole or several closely spaced holes with individuals sharing in the excavation process. We documented this process of collective excavation by establishing groups of 5–8 unfed first instar caterpillars in plastic portion-control containers having 32-mm diameter disks of cactus as described above. Each individual was uniquely marked with colored ink from a fine-tipped Sharpie© marker (Sharpie, Downers Grove, IL, USA); (Video S1). We saw no indication that marking in any way affected the behavior of the caterpillars. The activity of the caterpillars in the arena was video recorded in time lapse at the rate of 1 frame/second. These recordings were then analyzed beginning when the first attempt to penetrate the cladode was observed to determine the percentage of time each caterpillar spent: (1) actively excavating; (2) attempting to excavate but unable to because another caterpillar was excavating and blocking access to the hole; and (3) other activities, such as resting, moving about, or spinning silk. Observations of the aggregate continued until the groups had succeeded in excavating a full body length into the cladode. The study was replicated with eight different groups of caterpillars obtained from different egg sticks. Additional observations, however, were made of the five caterpillars in group 8 (Table 3) to determine the frequency with which the caterpillars brought excavated material to the surface and deposited it by defecation once the cohort was completely inside the cladode. Observations of each caterpillar began when it was first observed to exit the tunnel to defecate and continued for 35 successive bouts of defecation. Thus 175 discrete bouts of defecation were observed over the course of the study. The identical protocol of the study was also replicated seven additional times but with only a single caterpillar in each arena rather than groups of caterpillars. Since single individuals have little success in penetrating the cuticle of a cladode, as detailed below, the cuticle of the cladode was ruptured near the center with a small pin allowing volatiles from the softer tissue lying below the cuticle to escape and attract the caterpillar to the site. Recording of the frequency of activities of focal individuals for both studies was facilitated with the use of EthoLog^®^ software (Eduardo Ottoni, University of São Paulo, São Paulo, Brazil).

### 2.9. Role of Mandibular Gland Secretion in Pre-Excavation Aggregation

Previous studies showed that as the caterpillars move about they mark the substrate with a pheromone secreted from their mandibular glands which serves to maintain the aggregate by confining their movements to linear trails [8,9]. A study was conducted to test the hypothesis that the neonate caterpillars wander only short distances prior to and during the excavation process because they limit their activity to a small arena around the base of the egg stick that is marked with the pheromone. This was tested by spraying whole cladodes with the mandibular gland extract with the expectation that if the arena was defined by the mandibular gland secretion the caterpillars, rather than confining their activity to the base of the egg stick, would wander at random and independently over the whole of the cladode. The elongate mandibular glands extend the whole length of the body and their contents are readily extracted in hexane. To prepare the extract, 50 pairs of mandibular glands were dissected from 6th instar caterpillars following the procedure of [9]. The glands were ground in hexane and centrifuged. The supernate was drawn off and adjusted to 50 mL with additional hexane. Approximately 0.03 mL/cm^2^ of the solution was sprayed with a Preval© sprayer (Chicago Aerosol Co., Coal City, IL, USA) as a fine mist onto both sides of a section of a cladode (surface area = 163.4 ± 14.6 cm^2^) the cut ends of which were sealed with hot paraffin. After the solvent had evaporated, an egg stick within 48 h of hatching was attached to the cladode as described above. The behavior of the newly eclosed caterpillars was compared to their behavior on cladodes sprayed with hexane only as a control. This experiment was replicated 10 times with different egg sticks and different cladodes. Eggs sticks contained an average of 53.1 ± 1.7 eggs. Cladodes were maintained at approximately 21 °C at a mean RH of 76% in plastic boxes.

### 2.10. Trail Marking When the Cladode Cannot Be Excavated at the Eclosion Site

When the neonates are not able to penetrate the cuticle of the cladode at the base of the egg stick they are forced to move elsewhere. Although the caterpillars are known to mark and follow trails with their mandibular glands [8,9], the establishment of such trails by unfed, neonate caterpillars over the surface of the host plant has not been previously documented. Such behavior would serve to hold the travelling cohort and allow stragglers to locate their departed siblings. The behavior of caterpillars’ eclosing on a non-host substrate was documented for 10 colonies. For each replicate of the study, an egg stick was mounted at the center of a circular paper arena 14-cm in diameter. Concentric reference circles were marked on the paper at 1/2 cm intervals to facilitate tracing caterpillar movement patterns. The paper was placed in the bottom of a sealed petri plate underlain by a second plate containing a saturated solution of NaCl. Holes in the bottom of the upper plate allowed for communication between the two plates such that the air in the chamber containing the egg stick had an RH of approximately 76%. Hatching of the eggs and movement of the neonate caterpillars were monitored with two video cameras recording at the rate of one frame per 5 s interval. Two cameras were used to provide both a close up view of activity at the base of the egg stick and an overview of movement over the entire surface of the arena. Recording was continued until the caterpillars had constructed trails that led from the base of the egg stick to the edge of the arena. Data relevant to all aspects of eclosion, arena formation and dispersal were recorded and analyzed.

### 2.11. Data Analysis

Data were analyzed with Sigma Stat statistical software (Systat Software, San Jose, CA, USA) as reported below. All statistical error terms are reported as standard errors.

## 3. Results

### 3.1. Rate of Eclosion

**Figure 4:**
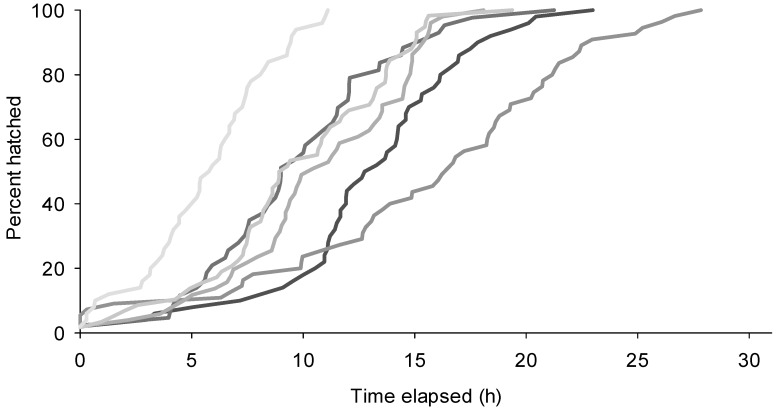
Relationship between time elapsed and percent of caterpillars’ eclosing from six different egg sticks.

When held at 21 ± 3 °C and 50%–70% RH, eclosion of caterpillars from an egg stick was markedly asynchronous with an average of 2.2 ± 0.23 eclosion events per hour (Figure 4). On average 20.1 ± 2.7 h elapsed between the eclosion of the first and last caterpillars from an egg stick.

### 3.2. Eclosion During Photo- and Scoto- Phases of a Light Cycle

For egg sticks held at 21 ± 3 °C and 15%–30% RH the per hour rate of eclosion events did not differ significantly between the dark phase (0.72 ± 0.16, *n* = 12 periods) and the light phase (1.14 ± 0.26, *n* = 14 periods; *t*-test with 24 df, *t* = 1.33, *p* = 0.2).

### 3.3. Effect of Relative Humidity on Egg Hatch

**Figure 5:**
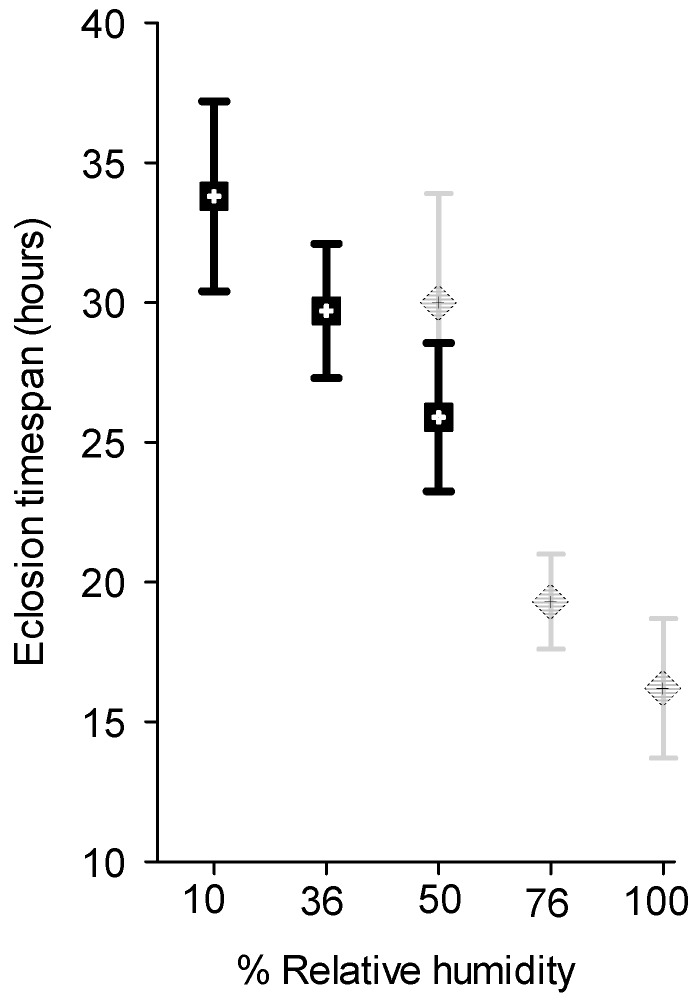
Time elapsed between the first and last caterpillars to eclose from sections of egg sticks held at different relative humidities. Light colored symbols are data points for study 1 and dark colored symbols for study 2.

Pettey [2] noted that when he covered a container containing egg sticks with a wet cloth to cool them down to slow hatching they hatched sooner. He attributed this to the softening of the egg shells by the moisture. The results reported here support this observation. For the first experiment, the time elapsed between the hatching of the first and last eggs at 100% RH was 16.2 ± 2.5 h, at 75% RH, 19.3 ± 1.7 h and at 50% RH, 30.0 ± 3.9 h. There was no significant difference between the 100% RH and 75% RH treatments, but both were significantly different from the 50% RH treatment (ANOVA *F* = 6.71, *p* = 0.006; Duncan’s Multiple Comparisons *p* < 0.05, Figure 5). The percentage of eggs that hatched did not differ among RHs, exceeding 90% for all treatments (Kruskal-Wallis ANOVA of Ranks on Arcsin Square Root transformations, *H* = 3.7, *p* = 0.16). In the second experiment, conducted at a lower range of RHs, time elapsed between the hatching of the first and last egg for egg sticks held at 50% RH was 25.8 ± 3.4 h, at 35% RH, 29.7 ± 2.4 h and at 10% RH, 33.8 ± 3.4 h (Figure 5). There was no significant difference among treatments (ANOVA, *F* = 1.62, *p* = 0.25). However, there was a marked increase in the number of eggs that failed to hatch at decreasing RH. The percentage of eggs that failed to hatch was 7.9% ± 0.04% when eggs were held 50% RH, 29.7% ± 0.08% when held at 35% RH and 45.1% ± 0.15% when held at 10% RH (ANOVA on Arcsin Square Root transformations, *F* = 3.6, *p* = 0.056).

### 3.4. Aggregation and the Initiation of Excavation

For egg sticks held at 21 ± 3 °C and 15%–30% RH, an average of 14.7 ± 2.8 h elapsed between the time the first caterpillar eclosed from an egg stick and the initiation of the excavation process. On average, 25.0 ± 4.1 caterpillars had eclosed at the time of the initiation of excavation and were aggregated at the excavation site (Table 1). The mean distance between the base of the egg stick and the site of initial excavation was 4.2 ± 0.86 mm. Once excavation was initiated, the aggregated caterpillars required 1.1 ± 0.13 h to excavate to the depth of one body length. There was no significant relationship between the number of caterpillars aggregated at the time excavation was initiated and the time it took to excavate to one full body length (Regression analysis, *R* = 0.23, *F* = 0.29, *P* = 0.61). When excavating, caterpillars initially chewed through the cuticle and tossed debris away from the opening with their mandibles. This often required considerable effort, with the caterpillars twisting the bodies from side to side to leverage their mandibles. After penetrating the cuticle they filled their guts with the softer tissue and defecated it in a ring shaped arena around the entrance site (Video S2). The mean distance from the egg stick to the edge of the arena was 10.7 ± 2.3 mm. Defecation behavior is described below.

### 3.5. Ability of Individual and Small Groups of Caterpillars to Penetrate a Cladode

Of the 61 caterpillars placed singly on circular disks of cactuses, 53 failed to penetrate the surface of the cactus. The remaining 8 caterpillars penetrated the surface of the cactus and were successful in feeding on the plant. In the replicates of the experiment with five caterpillars, all 13 of the groups successfully penetrated the surfaces of the plants and established feeding tunnels inside the cactuses. As shown in Table 1, between 13 and 41 caterpillars (mean = 25.0 ± 4.1) were aggregated at the base of egg sticks at the time excavation of a cladode was initiated, far in excess of the five caterpillars shown here to be able to successfully penetrate the plant. Although groups of five *Cactoblastis* neonates were not successful in penetrating cladodes in a study conducted by [7], groups of eight or more were. The difference between the results of their study and those reported here may be attributable to the relative toughness of the cuticle of the plants used in the two studies. In any case, the difference is small and both studies demonstrate that only a fraction of the total number of caterpillars that are typically aggregated at the time excavation are capable of initiating the excavation process.

### 3.6. Orientation of Neonates to Host Volatiles

In the olfactometer tests, 92.1% of the caterpillars entered the opening leading to the fragmented cactus (Table 2). The response was significantly greater than to the blank (3.7%) and to the water treatment (4.3%, Kruskal-Wallis one way ANOVA on ranks and Tukey’s subtest *H* = 18.65, *p* < 0.001, Video S3). There was no significant difference in the response to the blank and water treatments.

### 3.7. Group Excavation Behavior

**Figure 6:**
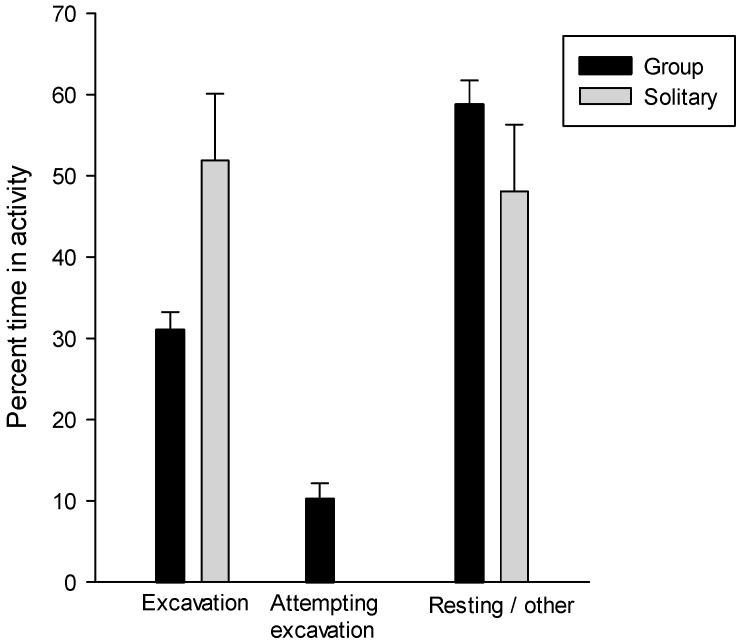
Percent of time spent in different activities by solitary and grouped caterpillars. See also Video S1.

Solitary individuals spent 51.9% ± 8.2% of their time excavating and 48.1% ± 8.2% in other activities (Figure 6 and Table 3). In groups of 5–8 caterpillars, individuals spent 31.04% ± 2.2% of their time excavating (range 20.4%–38.9%), 10.20% ± 1.9% attempting to enter the excavation (range 4.1%–19.4%), and 58.7% ± 3.0% moving about or resting (range 48.9%–74.3%, Table 4 and Figure 6). The difference in percent of time excavating was significantly different between the solitary and grouped caterpillars (Mann-Whitney Rank Sum Test *U* = 22.0, *p* = 0.03), but there was no significant difference between the sum of the percentages of time spent excavating and attempting to excavate by individuals in groups compared with the percent of time spent excavating by individuals (Mann-Whitney Rank Sum Test *U* = 9.0, *p* = 0.54).

All caterpillars in each group participated in excavating to some degree. An excavating caterpillar was frequently interrupted when another caterpillar tried to access the hole, often resulting in the displacement of the original caterpillar and resulting in an intermittency of excavation on the part of each caterpillar (Video S1). For example, the five caterpillars in group 8 (Table 4), engaged in a mean of 11.20 ± 0.66 discrete bouts of excavation, and 7.60 ± 0.51 attempts to reenter, punctuated by bouts of other activities (moving about, spinning and resting) over an observation period of 65 min.

The caterpillars show a pronounced, previously undescribed fixed action pattern associated with defecation. Initially, they defecate while the anterior part of their body is still inside the opening. Later they come completely out, head first, and move to the edge of the debris field where they appear to assess its location. They then turn, walk partway back to the entrance hole, then backup into the debris field and defecate (Video S2).

When the first instars are fully inside the cactus, their efforts are directed at tunneling, the rhythmic pace of which appears to exceed that which would be required to merely satisfy their nutritional needs. The five caterpillars in group 8 (Table 3) emerged from the mine to defecate at a mean interval of 9.48 ± 0.23 min then immediately returned to the task of mining, continuing in this manner non-stop over the course of the 35 successive bouts observed for each caterpillar. The interval of defecation varied within a remarkably narrow range among the five caterpillars (Table 5).

**Figure 7:**
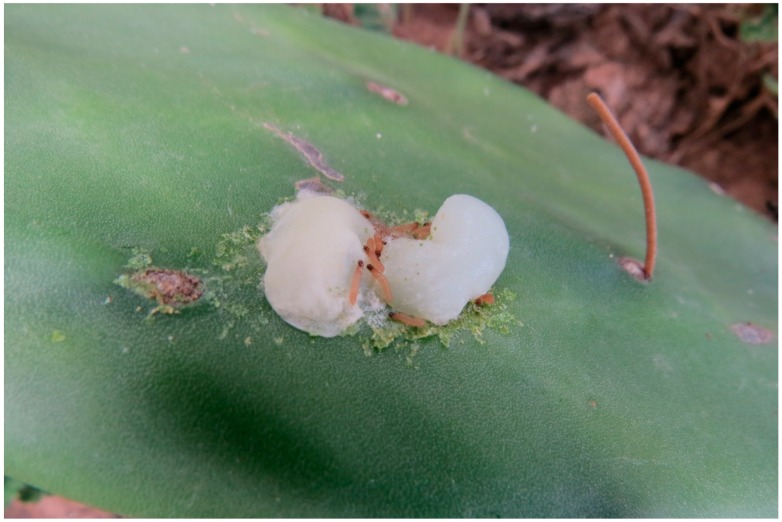
Larvae of *Cactoblastis cactorum* ejected from cactus by mucilaginous exudate.

It has been reported that the efforts of neonates in penetrating the cactus may be thwarted when their activity result in the expulsion of mucilaginous sap [2] (Figure 7). Only one instance of this was observed during the course of the present study and is documented here because the specific response of the neonates to the secretion has not been previously reported or detailed, and it illustrates the strong fidelity the caterpillars may show to the initial site of excavation. At the time of the event, documented in Video S4, two caterpillars were excavating side by side and had not yet penetrated a full body length into the cactus. Ten additional caterpillars were resting or spinning silk near the excavation site. When the eruption occurred, the two excavating caterpillars were forcefully ejected from the hole and engulfed in the viscid fluid which ran onto the surface and formed a puddle roughly 1.5-cm in diameter. Both caterpillars escaped by pushing their way through the exudate. Initially all of the non-involved caterpillars moved away from the exudate, but within a few minutes a few caterpillars began to imbibe liquid from the edges of the puddle. Eventually all 12 of the caterpillars joined in and continued to imbibe the liquid without rest. The imbibed liquid was deposited away from the excavation site gradually drawing down the puddle until the caterpillars were able to access the original entrance hole and continue to excavate. The total time required by the cohort to collectively remove the exudate was 156 min.

### 3.8. Role of Mandibular Gland Secretion in Pre-Excavation Aggregation

**Figure 8:**
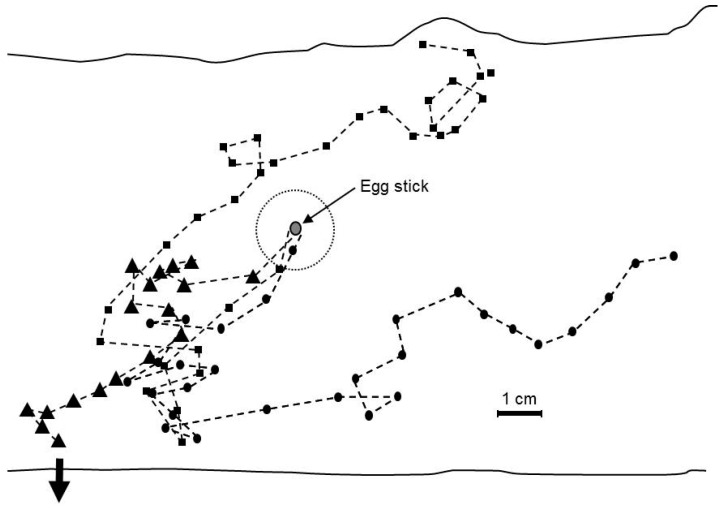
Dispersal pathways of the first three caterpillars to eclose on cladodes sprayed with mandibular gland extract. Circular dashed line encloses the silk-roofed arena. See also Video S5.

In all of 10 replicates of the experiment in which cladodes were sprayed with mandibular gland extract, neonate caterpillars failed to aggregate at the base of the egg stick, but dispersed independently over the surface of the cladodes soon after they eclosed (Figure 8, Video S5). Many of the isolated caterpillars lost purchase on the plant and were seen dangling from stands of silk before falling into the trays holding the cladodes. An average of 19.1 ± 3.0 caterpillars per colony were lost in this manner. No caterpillars were successful in entering the cladode in three of the replicates. For the other seven replicates, groups averaging 13.1 ± 2.9 caterpillars regrouped at sites 5.4 ± 0.38 cm from the base of their egg sticks and were successful in entering the plant. For all replicates of the controls, for which cladodes were sprayed with only hexane, the colonies aggregated at the base of their egg sticks, as illustrated in Video S6, and constructed their entrance holes 0.35 ± 0.08 cm from the base. There was a significant difference in the distance between the base of the egg stick and the entrance hole for the treatment and control replicates (Mann-Whitney Rank Sum Test *t* = 148, *p* < 0.001).

### 3.9. Trail Marking When the Cladode Cannot be Excavated Near the Eclosion Site

**Figure 9:**
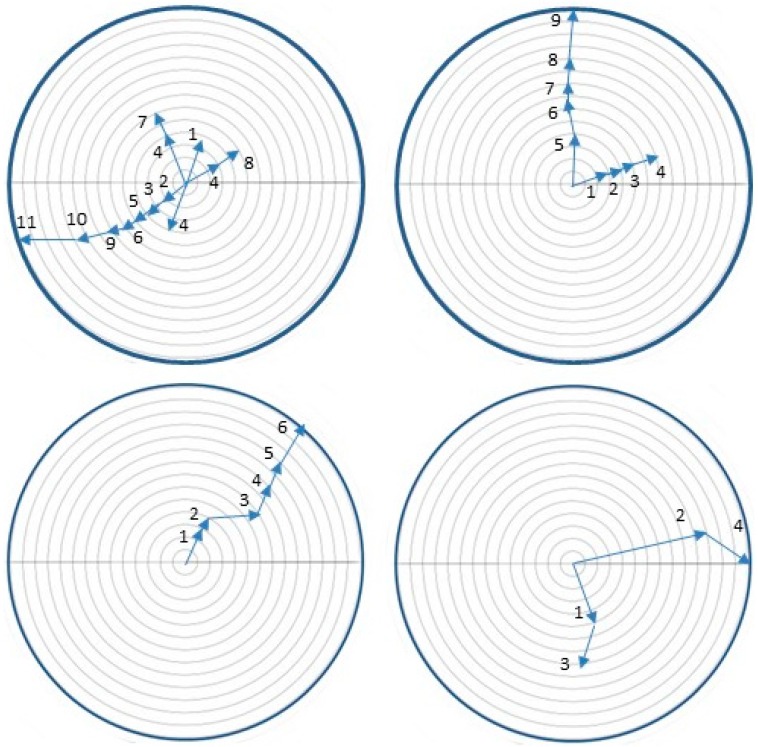
Representative examples of trail marking by colonies of *Cactoblasts cactorum* caterpillars abandoning egg sticks attached to paper arenas.

Neonates in all colonies established silk roofed arenas having a mean diameter of 1.7 ± 0.192 cm around of the base of the egg stick (Figure 1). The first concerted attempts to move off these arenas occurred when an average of 24.8 ± 2.8 caterpillar had eclosed and were assembled at the base of the egg stick, an average of 10.1 ± 0.88 h after the first caterpillars in the egg sticks had eclosed. At this time, a mean of 43.5% ± 3.5% of the eggs in an egg stick had hatched. These initial movements involved a collective effort by an average of 3.5 ± 0.34 caterpillars, the leader closely followed by the others, resulting in them establishing a trail that extended 1.7 ± 0.10 cm from the base of the egg stick before turning back. In three of the replicates, the initial trail was extended by subsequent waves of caterpillar and was the only trail that the colonies constructed before reaching the edge of the arena. For the remaining replicates, in addition to extending this initial trail, the caterpillars laid down other trails such that an average of 2.0 ± 0.37 trails were constructed per colony for all ten replicate of the study (Figure 9). On average, 4.5 ± 0.29 caterpillars participated in each wave of trail extension during which trails were extended 1.26 ± 0.35 cm before cohorts turned back. On average 7.5 ± 0.65 of these episodes of advancing then retreating occurred before a trail was extended to the edge of the arena, 6.1 ± 1.8 h after the first trail marking episode was initiated. Once a trail was established to the boundary of the arena, all of the caterpillars in the colony eventually moved over it and the colony aggregated along the edge of the arena.

## 4. Discussion

**Figure 10:**
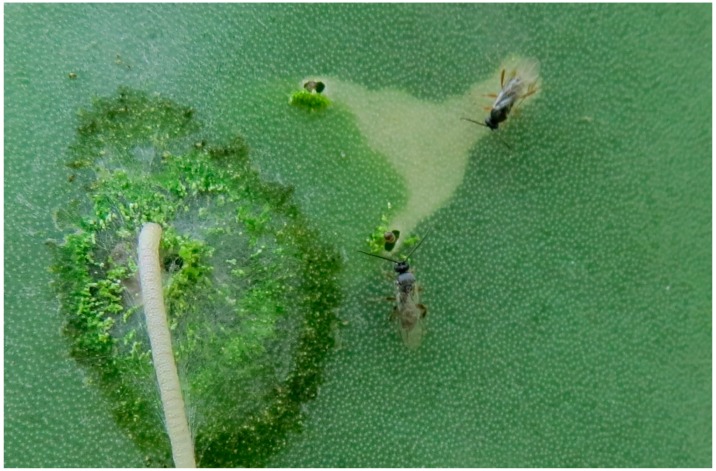
The parasitoid *Apanteles opuntiarum* (Hymenoptera: Braconidae) attacking first instar larvae of *Cactoblastis cactorum*.

This study shows that under laboratory conditions, eclosion of caterpillars from a shared egg stick is markedly asynchronous. McLean et al. [12], also conducted a laboratory study that showed that eclosion from a common egg stick occurs over a period of days. They reported that 90% percent of the eggs in egg sticks held at 20 °C hatched over a period of 9 days while 96% hatched over a period of 4 days when eggs were held at 30 °C. As they did not record the exact time of eclosion of eggs in eggsticks, or the RH at the time of testing, it is not possible to directly compare the results of their study with the present study. More general field-based observations were made by Dodd [1] and Pettey [2]. Both reported considerable variation in the time taken for all the eggs in an egg stick to hatch ranging from 3–4 h up to 2 to 3 days. Pettey [2] noted that the eggs of *C. cactorum* are laid at a rate of about one every 16 s, such that an egg stick containing 50 eggs would be completed in approximately 13 min. Thus, asynchronous hatching cannot be viewed as a simple consequence of the difference in the age of eggs. However, since the eggs in an egg stick differ in genetic makeup, it may not be surprising that the rate of embryogenesis would differ among eggs. If 25 days is taken as a typical time span between oviposition and the initiation of hatching, a difference of 20 h between the hatching of the first and last egg, as recorded in the present study, represents only a 3% difference in developmental time. Regardless of the cause of eclosion asynchrony, the phenomenon may be of some benefit to a colony since asynchronous hatching results in a reduction in the average amount of time that neonates are exposed to predators on the surface of the cactus. Ants, particularly in the genus *Cremastogaster*, and spiders have been reported to attack the exposed neonates [1,2,13]. The braconid, *Apanteles opuntiarum*, a specialist on *C. cactorum* [14] also attacks the newly eclosed caterpillars (Figure 10). In the present study, an average of 25.0 ± 4.1 caterpillars had eclosed at the time of the initiation of excavation and were aggregated at the excavation site (Table 1). Since there were an average of 50 caterpillars in our egg sticks, approximately 50% of the caterpillars were still within the protective envelope of the egg at the time their active siblings began the process of excavation, enabling the former to move directly from the egg to the interior of the cactus upon eclosion. As shown in Table 1 the neonates in our study were exposed on the surface of the plant for an average of 14.7 h before they began to excavate the plant.

Our study with distinctively marked caterpillars shows that in small groups, the excavation process is shared among all the siblings with each caterpillar working for a short period before another takes up the task. The interaction is remarkably cooperative with the actively excavating caterpillars readily yielding their places to others (Video S1). Although a single caterpillar initiated excavation in our studies, several caterpillars sometimes worked side by side to enlarge the opening (Video S1). No instances of overt aggressive behavior among siblings were observed during the process of collective excavation.

The response of caterpillars in the three-choice olfactometer shows that the caterpillars are strongly attracted to volatile components of the host that are released when the larvae chew into the plant and this is likely to account for the fact that the neonates excavate a single entrance hole or a few closely spaced entrance holes. Since they are already aggregated in a small arena at the base of the egg stick, they need only respond to olfactants over very short distances. However, a review of the videos of the performance of caterpillars in the olfactometers clearly shows that they are able to orient up odor gradients. As shown in Video S3, the caterpillars were strongly drawn to volatile components of the cactus and in some cases oriented directly to the opening from a distance exceeding 10 body lengths. The pronounced side to side pendulations of the anterior part of the caterpillar’s body while moving up an odor gradient is indicative of klinotaxis where comparisons of stimulus strength are made sequentially on either side of the insects body. The response results in the aggregated caterpillars directing their collective efforts toward excavating a common entrance hole. Dodd [1] observing the olfactory response of older caterpillars reported that “half-grown” caterpillars were attracted to whole, intact cladodes when 15 cm away but did poorly at distances in excess of 20 cm. Pettey [2] made similar observations.

Because of the watery nature of the host plant, the caterpillars of *C. cactorum* need to process large quantities of food to meet their need for energy and nutrients. Although there have been no studies to determine the assimilation efficiency of *Cactoblastis* when feeding on *Opuntia*, the tissue of the cactus on a per-unit-mass basis is relatively low in nutrients: 80%–95% of *Opunta ficus-indica* consists of water, 3%–7% of carbohydrate, and 0.5%–1% of protein [15]. Nonetheless, the very high frequency at which the tunneling caterpillars return, nonstop, to the surface to defecate during the early stages of excavation, as observed during the present study suggests that the enlargement of the of the cavern to a size adequate to accommodate the entire colony may take priority over the need to feed. It remains unclear, however, whether the caterpillars can efficiently digest highly water-diluted food with a feeding-defecation cycle of only 10 min. Moreover, the color and particulate consistency of the material that passes from the caterpillar’s hindgut suggest that it is little processed.

The dispersal of neonates from the base of the egg stick when cladodes were sprayed with mandibular gland extract indicates that the oil the caterpillars deposit as droplets as they move about not only serves as a trail marker, as previously reported [8,9], but also as an arena marker that functions to contain the neonates. The establishment of the arena begins when the newly eclosed caterpillars move from the egg stick onto the surface of the cactus. On the cactus, the caterpillars move hesitantly and turn back after venturing only short distances from their egg stick. In the process they lay down strands of silk and secrete droplets of mandibular gland fluid. Successive waves of newly eclosed caterpillars repeat the process until a silk-roofed arena is established around the base of the egg stick (Figure 1). The failure of the caterpillars to be contained within an arena but to disperse independently on sprayed cladodes is consistent to with the ability of the mandibular gland fluid to elicit a trail-following response. It is unclear, however, the extent to which physical or chemical properties of the silk component of the arena might also serve to contain the caterpillars since dispersal of the neonates on eclosion limits the buildup of silk at the base of the egg stick.

Our study shows that unfed neonates travel over the surface of the cactus *en masse* when they are unable to penetrate the cladode at their initial aggregation site at the base of the egg stick. The migration is facilitated by a trail pheromone that previous studies show is secreted from the mandibular glands [8,9]. Trail marking appears essential to colony cohesion during these over- the-surface forays. Although trail marking may also facilitate the surface movement of older colonies from cladode to cladode [1,2], trail marking may be particularly important to the neonates. A number of investigators have noted such dispersal under field conditions. Pettey [2] observed colonies attempting to enter cladodes at as many as six different locations after being repeatedly repelled by mucilaginous sap. Robertson and Hoffman [13] made similar observations noting that the larvae were sometimes not successful in gaining full entrance until the third stadium. Since the eclosion process is markedly asynchronous, trail marking appears essential to the maintenance of the cohort when early emerging caterpillars move away from the egg stick before the entire cohort has eclosed. The trail marker is water insoluble and non-volatile such that its communicative value persists long after being laid down [8,9].

## 5. Conclusions

The newly eclosed caterpillars of *C. cactorum* engage in suites of socially facilitated, adaptive behaviors that enable them to successfully colonize the interior of cactus cladodes. Caterpillars in the same egg stick did not eclose simultaneously but with a lapse of 20 h or longer, depending on the ambient RH. As caterpillars eclosed they aggregated *en masse* at the base of the egg stick in an arena typically less than 2-cm in diameter. No attempt to penetrate the plant occurred until, on average, approximately 25 neonates had aggregated, approximately 15 h after the first eggs hatched. If the cladode at the base of the egg stick could not be penetrated, caterpillars began to move off in an orderly search of another site approximately 10 h after eclosion began, leaving behind siblings in eggs that had not yet hatched. Both the initial aggregation of neonates at the site of the egg stick and the ability of caterpillars that eclose late to locate cohorts of departed siblings are attributable to pheromonal components of the caterpillar’s mandibular glands that are used to mark both arenas and trails. When the cuticle of the host was penetrated by a caterpillar the cohort showed strong attraction to volatile host chemicals that emanated from the opening and mounted a shared effort involving discrete bouts of excavating and rest on the part of individuals to deepen the opening until they reach the interior. Once inside the plant, the caterpillars adopted a regular rhythm of defecating, returning to the surface approximately every 10 min to deposit material mined from the soft inner body of the cladode.

## Figures and Tables

**Table 1:** Eclosion and initiation of excavation behavior by neonates of *Cactoblastis cactorum*.

Colony	Number of Caterpillars Eclosed when Excavation Initiated	Time Elapsed between First Eclosion and Initiation of Excavation (HR)	Time between Initiation of Excavation Until One Body Length Penetration (HR)
1	13	9.4	1.4
2	38	15.4	1.4
3	41	11.0	1.0
4	18	15.7	1.0
5	26	23.9	0.7
6	16	3.9	1.6
7	23	23.6	0.8
Mean ± SE	25.0 ± 4.1	14.7 ± 2.8	1.1 ± 0.13

**Table 2:** Response of the neonate caterpillars of *Cactoblastis cactorum* in olfactometer tests.

Replicate	Instar	Number of Caterpillars	Duration of Test (min)	Number of Choices for Treatment		
				Blank	Water	Cactus
1	Fed 1st	31	28	0	0	31
2	Fed 1st	29	88	0	1	28
3	Fed 1st	13	37	0	1	12
4	2nd–3rd	15	52	3	0	10
5	2nd	20	79	0	2	15
6	Unfed 1st	19	52	0	0	17
7	Unfed 1st	17	33	0	0	14
8	Unfed 1st	11	33	1	2	6
9	Fed 1st	20	24	3	0	18
Mean ± SE				0.79 ± 0.44	0.67 ± 0.29	16.78 ± 2.70

**Table 3:** Percent of time spent excavating and in other activities by solitary neonates of *Cactoblastis cactorum*.

Replicate	Mean Percent of Time		Episodes	
	Excavating	Other	Excavating	Other
1	36.1	63.9	12	23
2	74.3	25.7	8	7
3	65.0	35.0	18	21
4	80.0	20.0	11	10
5	40.8	59.2	38	40
6	21.1	78.9	66	112
7	45.8	54.2	33	43
Mean ± SE	51.9 ± 8.2	48.1 ± 8.2	26.6 ± 7.9	36.6 ± 13.6

**Table 4:** Percent of time spent excavating and in other activities by groups of neonates of *Cactoblastis cactorum*.

Group	Mean Percent of Time *		
	Excavating	Attempting to Excavate	Other
1	31.8	19.4	48.9
2	29.0	17.7	53.3
3	20.4	5.2	74.3
4	35.9	4.1	60.0
5	37.0	9.3	53.7
6	38.9	8.6	52.5
7	25.3	7.8	67.0
8	30	9.5	60.3
Mean ± SE	31.0 ± 2.2	10.2 ± 1.9	58.7 ± 3.0

* Each value is the mean for the group of 5–8 caterpillars.

**Table 5:** Interval between successive episodes of defecation for five first instar caterpillars of *Cactoblastis cactorum*. N = 35 successive episodes for each caterpillar.

Caterpillar	Mean ± SE Interval between Successive Bouts of Defecation
1	8.5 ± 0.49
2	10.4 ± 0.42
3	11.7 ± 0.48
4	10.2 ± 0.48
5	8.8 ± 0.36
Mean ± SE *	9.48 ± 0.23

* derived from raw data of 175 data points.

## Supplementary Materials

The following are available online at www.mdpi.com/2075-4450/7/4/59/s1. Video S1: Collective excavation behavior of marked caterpillars, Video S2: Excavation and defecation; Video S3: Response of caterpillars in olfactometer; Video S4: Response of caterpillars to mucilage; Video S5: Dispersal of caterpillars on treated cladodes; Video S6: Aggregation of caterpillars on control cladodes.

## References

[1] Dodd A.P. (1940). The Biological Campaign against Prickly Pear.

[2] Pettey F.W. (1947). The biological control of prickly pears in South Africa Science Bulletin. Dept. Agric. Union S. Afr..

[3] Zimmermann H.G., Moran V.C., Hoffmann J.H. (2000). The renowned cactus moth, *Cactoblastis cactorum*: Its natural history and threat to native *Opuntia* floras in Mexico and the United States of America. Divers. Distrib..

[4] Legaspi J.C., Baez I., Legaspi B.C. (2009). Phenology and egg production of the cactus moth, *Cactoblastis cactorum* (Lepidoptera: Pyralidae): Comparison of field census data and life stage development in the field. J. Entomol. Sci..

[5] Robertson H.G. (1985). The Ecology of *Cactoblastis cactorum* (Berg) (Lepidoptera: Phycitidae) in Relation to Its Effectiveness as a Biological Control Agent of Prickly Pear and Jointed Cactus in South Africa. Ph.D. Thesis.

[6] Hoffmann J.H., Zimmermann H.G., Delfosse E.S. (1989). Ovipositional and Feeding Habits in Cactophagous Pyralids: Prediction for Biological Control of Cactus Weeds in South Africa. Proceedings of the VII International Symposium on Biological Control of Weeds.

[7] Varone L., Manteca Acosta M., Logarzo G.A., Briano J.A., Hight S.D., Carpenter J.E. (2012). Laboratory performance of *Cactoblastis cactorum* (Lepidoptera: Pyralidae) on South and North American *Opuntia* species occurring in Argentina. Fla. Entomol..

[8] Fitzgerald T.D., Wolfin M., Rossi F., Carpenter J.E., Pescador-Rubio A. (2014). Trail marking by larvae of the cactus moth, *Cactoblastis cactorum*. J. Insect Sci..

[9] Fitzgerald T.D., Kelly M., Potter T., Carpenter J.E., Rossi F. (2015). Trail following response of larval *Cactoblastis cactorum* to 2-Acyl-1,3 cyclohexanediones. J. Chem. Ecol..

[10] Wilson E.O. (1971). The Insect Societies.

[11] Peterson A. (1934). Entomological Techniques: How to Work with Insects.

[12] Mclean S.C., Bloem K.A., Bloem S., Hight S.D., Carpenter J.E. (2006). Effect of temperatures and length of exposure time on percent egg hatch of *Cactoblastis cactorum* (Lepidoptera: Pyralidae). Fla. Entomol..

[13] Robertson H.G., Hoffmann J.H. (1989). Mortality and life-tables of *Cactoblastis cactorum* (Berg) (Lepidoptera: Pyralidae) compared on two host-plant species. Bull. Entomol. Res..

[14] Varone L., Logarzo G., Martinez J.J., Navarro F., Carpenter J.E., Hight S.D. (2015). Field host range of *Apanteles opuntiarum* (Hymenoptera: Braconidae) in Argentina, a potential biocontrol agent of *Cactoblastis cactorum* (Lepidoptera: Pyralidae) in North America. Fla. Entomol..

[15] Ginestra G., Parker M.L., Bennett R.N., Robertson J., Mandalari G., Narbad A., Lo Curto R., Bisignano G., Faulds C., Waldron K. (2009). Anatomical, chemical, and biochemical characterization of cladodes from prickly pear [*Opuntia ficus-indica* (L.) Mill.]. J. Agric. Food Chem..

